# The Local Inflammatory Responses to Infection of the Peritoneal Cavity in Humans: Their Regulation by Cytokines, Macrophages, and Other Leukocytes

**DOI:** 10.1155/2012/976241

**Published:** 2012-02-26

**Authors:** Marien Willem Johan Adriaan Fieren

**Affiliations:** Department of Internal Medicine, Erasmus Medical Center, Centrumlocatie, P.O. Box 2040, 3000 CA Rotterdam, The Netherlands

## Abstract

Studies on infection-induced inflammatory reactions in humans rely largely on findings in the blood compartment. Peritoneal leukocytes from patients treated with peritoneal dialysis offer a unique opportunity to study in humans the inflammatory responses taking place at the site of infection. Compared with peritoneal macrophages (pM*ϕ*) from uninfected patients, pM*ϕ* from infected patients display *ex vivo* an upregulation and downregulation of proinflammatory and anti-inflammatory mediators, respectively. Pro-IL-1*β* processing and secretion rather than synthesis proves to be increased in pM*ϕ* from infectious peritonitis suggesting up-regulation of caspase-1 *in vivo*. A crosstalk between pM*ϕ*, *γδ* T cells, and neutrophils has been found to be involved in augmented TNF*α* expression and production during infection. The recent finding in experimental studies that alternatively activated macrophages (M*ϕ*2) increase by proliferation rather than recruitment may have significant implications for the understanding and treatment of chronic inflammatory conditions such as encapsulating peritoneal sclerosis (EPS).

## 1. Introduction

Continuous ambulatory peritoneal dialysis (CAPD) was introduced in 1978 as a new treatment modality for patients with end-stage renal failure. In CAPD, after infusion of typically 2 litres of dialysis fluid via a catheter into the peritoneal cavity, retained metabolites diffuse from the blood to the peritoneal cavity during a dwell time of 4 to 8 hours, after which the dialysis fluid is drained and replaced with fresh dialysis fluid. In this way, the patient exchanges 3–5 times a day dialysis fluid. A major complication of CAPD is peritonitis caused by contamination by microorganisms that can enter the peritoneal cavity via infusion of dialysis fluid during the exchange, or by spreading of an infection from the skin and tissue around the catheter to the peritoneal cavity, or from the intestines [1]. In the early years, an episode of peritonitis occurred on average one time per 8 treatment months, but since the nineties the frequency was substantially reduced to one time every 24 months due to novel connections of the infusion systems. These so-called “flush-before-fill” systems reduce the risk of peritonitis during the exchange of dialysis fluids, which is caused especially by coagulase negative Staphylococci and other gram positive microorganisms. Infectious peritonitis is characterized by abdominal pain and turbid drained dialysate (peritoneal effluent) due to an increased number of leukocytes more than 50% of which are neutrophilic PMN's. Peritonitis is almost invariably revealed by opalescence of dialysate, which is noticed by patients when the leucocyte count is greater than 100/mm^3^. The majority of peritonitis episodes can be treated successfully with the intraperitoneal administration of antibiotics while continuing CAPD.

Infectious peritonitis in CAPD patients has been shown to provide a unique opportunity to study the inflammatory reactions in humans at the site of inflammation by studying cellular players including macrophages, lymphocytes, granulocytes, and mesothelial cells as well as soluble mediators present in peritoneal effluent [2–4]. In this paper, various studies are reviewed that are conducted in the past few decades on this topic with emphasis on the role of macrophages (M*ϕ*) and cytokines. The findings will be put in the context of new insights that developed the past decade in the biology of M*ϕ* and cytokines. Studying leukocytes from an inflammatory environment can make a valuable contribution to a better understanding of inflammatory reactions in humans.

## 2. Macrophages, Heterogeneity versus Polarization

**Figure 1:**
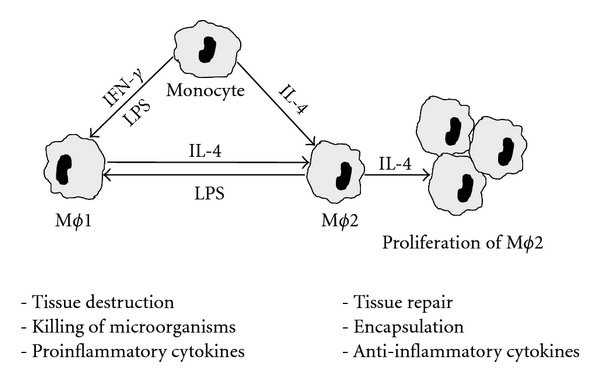
Simplified model of the paradigm of polarized macrophage activation. Monocytes are either classically (M*ϕ*1) or alternatively (M*ϕ*2) activated. Substances such as IFN-*γ* and LPS induce classical activation while IL-4, IL-13, and TGF*β* cause alternative activation. M*ϕ*1 macrophages typically produce high levels of proinflammatory proteins including TNF*α*, IL-1*β*, IL-12, and IL-23 as well as reactive oxygen and nitrogen intermediates. M*ϕ*2 produce high levels of the anti-inflammatory cytokines IL-10 and IL-1ra and show a high expression of scavenger and mannose receptors. Under the influence of IL-4, M*ϕ*2 can accumulate in tissues by local proliferation rather than by recruitment. Either functional phenotype can shift to another depending on microenvironmental influences, speaking against rigid interpretation of the dichotomy between the macrophage phenotypes.

Tissue M*ϕ* are derived from circulating blood monocytes, which in turn arise from their bone marrow precursors. These cells together make up the mononuclear phagocyte system, as described by van Furth and Cohn [5]. After monocytes have entered the tissues to become M*ϕ*, they have the potential to acquire a variety of different functional attributes depending on signals they receive from the environment. Thus, the mononuclear phagocyte system consists of a heterogeneous and highly versatile, multipotential cell population. The differentiation and activation to diverse functions in the tissues are governed by the presence of regulatory signals in the environment and occur in several distinct steps [6]. In the past decade, a new view on M*ϕ* differentiation and activation has been developed. *In vitro* two types of M*ϕ* are distinguished: Classically activated M*ϕ* display a pro-inflammatory profile, induced by IFN-*γ* or LPS, whereas alternatively activated M*ϕ* express anti-inflammatory and tissue repair properties induced by IL-4 or IL-13 [7–11]. IFN-*γ* is a prototypical Th-1 cell secretory product, while IL-4 and IL-13 are produced by Th-2 cells and M*ϕ*. Classically and alternatively activated M*ϕ* are also named as M*ϕ*1 and M*ϕ*2, mirroring the Th-1 and Th-2 polarization, respectively. Type 1 and type 2 inflammation represent ancient innate pathways with fundamentally different purposes. Type 1 promotes killing of microbial pathogens and intracellular parasites and is involved in tissue destruction and tumor resistance. Type 2 participates in tissue repair and controls infection with macroparasites through encapsulation. M*ϕ*1 typically show a high expression of the cytokines IL-12, IL-23, TNF*α*, IL-1*β*, and M*ϕ*1 chemokines and are efficient producers of reactive oxygen and nitrogen intermediates, whereas IL-10 production is low. In contrast, in M*ϕ*2 expression of IL-12, IL-23, TNF*α*, and IL-1*β* is low, whereas expression of IL-10, IL-1ra, TGF*β*, M*ϕ*2 chemokines and scavenger, mannose and galactose receptors is high. In experimental *in vivo* studies, it has been found that a subset of patrolling, circulating monocytes, which may correspond to human CD16+ monocytes, are rapidly recruited to the peritoneal cavity, peaking at 2 hours after infection with Listeria monocytogenes, when PMN is only beginning to enter the peritoneal cavity [12]. After 1 and 2 hours after infection these mononuclear phagocytes produce TNF*α* and show an upregulated expression of genes coding for IL-1 and various chemokines and pattern recognition receptors such as toll-like receptors (TLRs). Notably, the production of TNF*α* and IL-1*β* is transient and turns off at 8 hours, whereas these mononuclear phagocytes turn on, at 2 and 8 hours, in genes involved in tissue remodeling. A different subset of conventional monocytes arrive later and give rise to inflammatory dendritic cells (DCs) and M*ϕ*1 macrophages [8, 12]. In a recent experimental study, it was found that both resident and recruited M*ϕ* can be alternatively activated and be driven to proliferate *in situ* by a Th-2 environment *in vivo*, implying that there is neither a specific precursor for M*ϕ*2 nor is proliferative capacity restricted by lineage [13]. While the paradigm of macrophage dichotomy is well established, employing it as a rigid scheme could bring about a risk of oversimplification. Thus, M*ϕ* can reversibly shift their functional phenotype through a multitude of patterns in response to changes in cytokine environment, as illustrated in Figure 1 [14]. In humans, arginase, which is considered to be characteristic of alternatively activated macrophages, is not expressed prominently IL-4-induced M*ϕ*2 macrophages [15]. Furthermore, during the resolution phase of experimental inflammation a M*ϕ* phenotype with properties of both M*ϕ*1 and M*ϕ*2 could be distinguished [16].

## 3. Peritoneal Macrophage (pM*ϕ*) from CAPD Patients

Approximately 1–40 millions of leukocytes can be collected from peritoneal effluent after a dwell time of 6–8 hours. The yield decreases in the course of CAPD treatment. In uninfected patient, the leukocyte population was found to be composed of 85% mononuclear phagocytes by nonspecific esterase staining, while >75% of each cell population was HLA-DR+. Six percent were neutrophilic and/or eosinophilic PMNs [17, 18]. Using flow cytometric analysis for surface markers, about 40% of the peritoneal cells were identified as lymphocytes [19]. Various subsets are distinguished in peritoneal lymphocytes including B cells and various subsets of T cells. Two to six percent of peritoneal cells can be characterized as DC's, which are differentiated in the peritoneal cavity from monocyte-derived CD14+ cells [20, 21]. Peritoneal CD14+ cells were characterized as M*ϕ*2 macrophages on the basis of a CD163+CD16− phenotype, a high capacity for phagocytosis and production of high amounts of IL-10, sharing these properties with *in vitro *polarized M*ϕ*2 [19]. In contrast, the production of substantial amounts of IL-6, as found in this study, is a property of M*ϕ*1 rather than M*ϕ*2. The CAPD cell population is continually renewed and is exposed to dialysis fluids with an unphysiological composition and to the dialysis catheter. Yet, in many respects the macrophages from CAPD patient bear resemblance to those from healthy women undergoing laparoscopy [17, 18]. Compared with such cells from rats, CAPD pM*ϕ* resemble starch-elicited rather than resident cells [22].

When a peritoneal infection becomes clinically manifest, there is a sharp, up to 100 fold increase in peritoneal leukocytes, 50–90% of which are neutrophils. Also the number of pM*ϕ*, dendritic cells and various subsets of lymphocytes show a marked increase, including *γδ* T cells [20, 23].This minisubset is rapidly recruited to the inflammatory site and responds to the microbial molecule HMB-PP that is found in various species—30% to 50% of peritonitis episodes is caused by HMB-PP+ microbes—and is released when microorganisms are killed by other leukocytes including neutrophils [24]. By interaction of *γδ* T cells with mononuclear phagocytes, the inflammatory reaction is amplified. Already one to two days before the infection becomes clinically manifest, an increased number of pM*ϕ* and neutrophils is found [25]. Following appropriate antibiotic treatment, the mononuclear cells and especially the neutrophils show a sharp drop in the next few days, resulting in a relative increase of pM*ϕ* and lymphocytes. While on the first day of the peritonitis pM*ϕ* outnumber lymphocytes, in the resolution phase the macrophages/lymphocytes ratio is reversed [26]. Using flow cytometry, pM*ϕ* from infected patients displayed an increased expression and production of selected M*ϕ*2-associated cell surface markers (CD163+) and chemokines (CCL18), respectively, but expression of the M*ϕ*2-associated mannose receptor CD206+ was lower in peritonitis pM*ϕ*. Gene expression of TGF-*β*1, metalloproteinase 9 (MMP9), and CCL18 in pM*ϕ* from infected and uninfected patients were similar [27].

## 4. Cytokines in CAPD during Infectious Peritonitis

The pro-inflammatory cytokines IL-1*β*, TNF*α*, and IL-6 play a key role in the inflammatory response. By exerting their pleiotropic effects in an autocrine, paracrine, and endocrine fashion, these cytokines are able to orchestrate the inflammatory responses. Although they can be produced by various cells, macrophages are the prototypical cell source. PM*ϕ* from CAPD patients collected during infectious peritonitis, showed a marked increase in the secretion of TNF*α* and IL-1*β* as compared with macrophages from infection free patients, when they were stimulated *ex vivo* with LPS [28, 29]. In contrast, unstimulated pM*ϕ* secreted similar amounts of TNF*α* and IL-1*βex vivo* in pM*ϕ* from patients with and without infection. These findings are in line with the paradigm of stepwise activation of M*ϕ*. On the other hand, the *ex vivo* secretion of the anti-inflammatory IL-10 was decreased in peritonitis macrophages, in line with a pro-inflammatory phenotype [30]. In the effluent from patients with infectious peritonitis, as compared with uninfected patients, increased levels of various pro-inflammatory cytokines were found, including IL-1*β*, IL-8, TNF*α*, IL-6, and IFN*γ* [26, 31–35]. Remarkably, also levels of anti-inflammatory cytokines for example, TGF*β* and IL-1ra were elevated [26, 32, 36]. It should be noted that in addition to M*ϕ* and other leukocytes, mesothelial cells may also contribute substantially to the production of various cytokines including IL-6 and IL-8 [37, 38]. 

**Figure 2:**
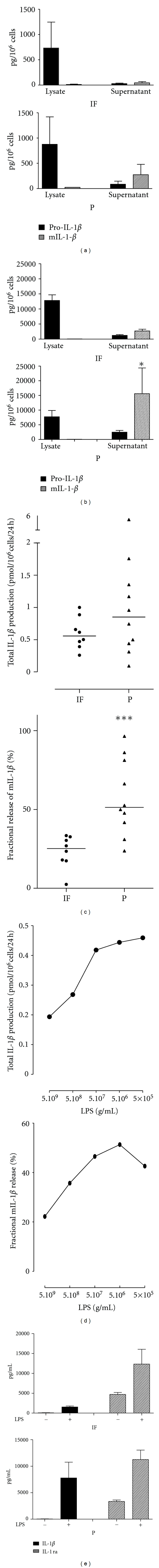
Peritoneal macrophages (pM*ϕ*) were isolated from CAPD patients who were at least 3 months infection free (IF), or during an episode of infectious peritonitis (P), prior to the start of treatment with antibiotics. 1.10^6^ Cells were incubated *ex vivo* during 24 h in medium or 5 *μ*/mL of LPS. Cytokines were determined in supernatants or cell lysates using ELISA. (a) Pro-IL-1*β* production and processing and mIL-1*β* release. Quantities of pro-IL-1*β* (black bars) and mature IL-1*β* (mIL-1*β*) (white bars), expressed in pg/10^6^ cells/24 h, using ELISA specific for either form of IL-1*β* (CISTRON^R^), in supernatants and cell lysates from pM*ϕ* isolated during IF-period (*n* = 8) or P (*n* = 10) and incubated in medium alone (a) or 5 *μ*/mL of LPS (b). Values are expressed as (M ± SEM). mIL-1*β* levels in supernatants from LPS-stimulated pM*ϕ*s were significantly higher when cells were isolated during P (*P* < 0.05), while pro-IL-1*β* in cell lysates was decreased (*P* ≈ 0.05). (c) total IL-1*β* production (= pro-IL-1*β* + mIL-1*β* in supernatants + cell lysates) expressed in pmol/10^6^ cells/24 h for each IF-period (circles) and P episode (triangles) in LPS stimulated pM*ϕ*. Difference between total IL-1*β* production from IF and P pM*ϕ* was not statistically significant. fraction of total IL-1*β* that is released in supernatant as mIL-1*β*, expressed as percentage. Fractional release from peritonitis pM*ϕ* was significantly higher (*P* < 0.005). (d) total IL-1*β* production and fractional mIL-1*β* release in response to increasing doses of LPS in pM*ϕ* isolated during 3 episodes of peritonitis, expressed in pmol/10^6^ cells/24 h. Total IL-1*β* production increased in a dose-dependent fashion. Within the range of 5.10^−9^–5.10^−6 ^g/mL, LPS induced a dose-related increase in fractional IL-1*β* release. (e) release of IL-1*β* and IL-1ra in supernatants from infection-free (*n* = 15) and peritonitis pM*ϕ* (*n* = 8) stimulated with and without 5 *μ*g/mL of LPS. Substantial amounts of IL-1ra were released in unstimulated cells. LPS-stimulated peritonitis pM*ϕ* released similar quantities of IL-1*β* and IL-1ra.

**Figure 3:**
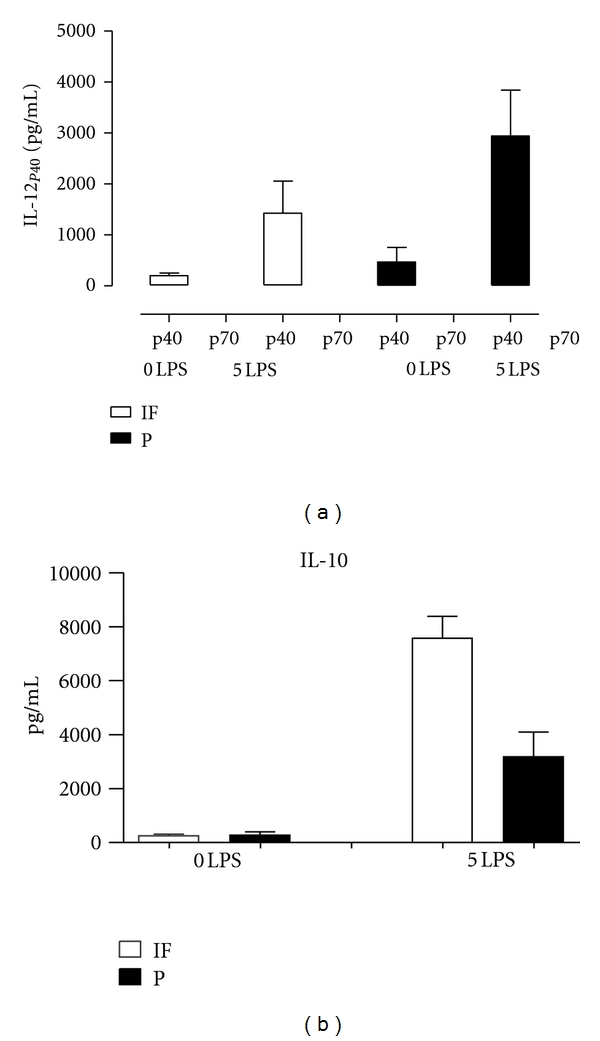
(a) Patients and methods as in Figure 1. *Ex vivo* IL-12_p40_ release is stimulated by LPS. Difference between pM*ϕ* from an infection-free period (*n* = 8) and an episode of peritonitis (*n* = 8), was statistically not significant. Using an ELISA specific to the bioactive, hetero-dimeric IL-12_p70_, no active IL-12 was detectable in supernatants from uninfected patients (*n* = 5) nor in those with peritonitis (*n* = 5), whether or not the cells were stimulated with LPS. Consistent with this finding, virtually no IL-12_p35_ mRNA was expressed (data not shown). All peritonitis episodes were caused by gram positive bacteria. (b) *ex vivo* IL-10 release from pM*ϕ* from infected patients (*n* = 8) is decreased as compared with pM*ϕ* from an infection free environment (*n* = 8), (*P* < 0.01). IL-10 levels in peritoneal effluents from peritonitis were higher compared with infection-free effluents, 235 pg/mL and 25 pg/mL, respectively (data not shown in figure).

We investigated at which level the increased capability of peritonitis pM*ϕ* to secrete IL-1*β* after *ex vivo* stimulation with LPS occurs, using ELISA's specific to the 32 kDa, biologically inactive pro-IL-1*β* and the mature 17 kDa, bioactive IL-1*β* [39]. Pro-IL-1*β* processing and subsequent release of mature IL-1*β* (mIL-1*β*) rather than its production were found to be increased in peritonitis pM*ϕ* (Figures 2(a), 2(b), and 2(c)), suggesting increased caspase-1 activity. Caspase-1 is present in the cell as the bioinactive pro-caspase-1 to become a bioactive cysteine protease after autocleavage. In the last decade, the understanding of the molecular mechanisms behind caspase-1 activation has been significantly increased. Briefly, NOD-like receptors (NLRs), present in the cytosol, recognize microbial molecules leading to oligomerization of NLRs and along with recruited pro-caspase-1 and other proteins, to the forming of multiprotein inflammasome complexes [40]. This results in auto-cleavage and activation of caspase-1, whereupon pro-IL-1*β* is cleaved and mIL-1*β* is released by an unconventional, poorly understood, mechanism as IL-1*β* lacks a signal peptide [41]. Microbial ligands induce transcription of pro-IL-1*β* and inflammasome components by activation of the transmembrane TLRs. Taken together, in the setting of our study increased caspase-1 activation might be postulated as priming mechanism *in vivo*. Interestingly, in a study using high-density oligonucleotide microarrays to investigate the transcriptional profile induced in human monocytes by IL-13, one the most striking findings, besides a variety of other characteristic genetic markers of alternatively activated macrophages, was downregulation of caspase-1 and changes in other components of the IL-1 system such as up-regulation of IL-1ra [15]. The LPS-inducible caspase-1 activity was also found to be reduced, resulting in a decrease in pro-IL-1*β* processing. Further studies are needed to reveal which molecular mechanisms account for the increased IL-1*β* processing and export in peritonitis pM*ϕ*. We also found that LPS stimulated not only pro-IL-1*β* production but also release of mIL-1*β* in a dose-dependent fashion, suggesting a stimulating effect of LPS on caspase-1 activity. (Figure 2(d)) PM*ϕ* displayed a rather high constitutive production of IL-1ra that further increased by stimulation with LPS, with pM*ϕ* from infection-free and peritonitis patients releasing similar amounts (Figure 2(e)). It has been reported that a 10–500 fold molecular excess of IL-1ra is required to obtain 50% inhibition of IL-1 biological effects *in vitro* [42]. In our study, similar amounts of IL-1ra and IL-1*β* were released in LPS-stimulated peritonitis pM*ϕ* implying a virtually unimpeded secreted IL-1 bioactivity. There was no production of the bioactive form of IL-12 (Figure 3(a)). The secretion of the anti-inflammatory cytokine IL-10 by LPS-stimulated peritonitis pM*ϕ* was significantly reduced (Figure 3(b)). However, IL-10 levels in peritoneal effluent were higher during peritonitis. The large increase in macrophages and other leukocytes during peritonitis, probably accounts for the discrepancy in the direction of the changes of IL-10 and other anti-inflammatory cytokines between macrophage cultures and peritoneal effluents. Absorption of pro- and anti-inflammatory cytokines from the infectious inflammatory site might offer in part an explanation for the discrepancy in the blood compartment between higher levels of circulating pro-inflammatory cytokines and a decreased capacity of blood monocytes to secrete TNF*α* and IL-1*β* as found in patients with sepsis. Compartmentalization of the inflammatory response is a key feature of the sepsis syndrome [43].

PM*ϕ* from infected patients have also an increased capability to release TNF*α* [29]. PGE_2_ has been found to have strongly inhibitory effects on LPS-stimulated TNF*α* release, almost eliminating the actions of LPS in a clearly dose-related fashion, whereas cyclooxygenase inhibition caused an increase in TNF*α* release [44]. The PGE_2_-induced downregulation, which was similar for pM*ϕ* from an infectious or infection-free environment, is probably brought about via elevation of intracellular cAMP levels. Moreover, it has been found that peritonitis macrophages have suppressed cAMP levels and a diminished release of prostaglandins compared to uninfected macrophages [45, 46]. Similarly, *ex vivo* stimulation of pM*ϕ* from uninfected patients with *Staphylococcus epidermidis* induced a marked decrease of cyclooxygenase products [47]. Prostaglandins are known for their pro-inflammatory effects, notably on the vascular components of inflammatory reactions, but in various settings these short-lived and locally acting substances have proved to possess anti-inflammatory properties as well. Recently it was reported, that, using low-dose and high-dose zymosan induced peritonitis as a model for self-limiting, resolving inflammation, and a more protracted response leading to systemic inflammation, respectively, pM*ϕ* from either environment displayed distinct characteristics [16]. PM*ϕ* from the protracted peritonitis had a typical M*ϕ*1 phenotype, while those from the resolving inflammation had characteristics of both M*ϕ*1 and M*ϕ*2 and were named as resolving macrophages (rM*ϕ*). These rM*ϕ*, as compared with M*ϕ*1, released *ex vivo* fewer pro-inflammatory cytokines, including TNF*α*, IL-1*β*, and IL-12 but more IL-10 and PGD_2_. The expression of COX 2, iNOS, and intracellular cAMP contents were also increased. Elevating cAMP levels by cAMP analoga transformed M*ϕ*1 to r*ϕ*M, whereas cAMP inhibitors converted rM*ϕ* to M*ϕ*1. These findings demonstrate that cAMP plays a central role in the regulation of M*ϕ* phenotype. In addition, it has been found that cyclooxygenase inhibition improved bacterial killing and resistance to infection in mice and humans, confirming the important role of cAMP. Interestingly, COX 1 rather than COX 2 turned out to be the predominant form that is active during infection [48]. Similarly, phagocytosis of apoptotic cells by M*ϕ* proved to inhibit the production of several mediators such as IL-1*β*, TNF*α*, and IL-10, but it increased the production of TGF-*β*1, PGE_2_, and PAF [49]. The latter mediators induced suppression of LPS-stimulated cytokine production by such M*ϕ*. In contrast, indomethacin restored the inhibition of cytokines and inhibited TGF-*β*1 production by phagocytosing M*ϕ*. These findings show that PGE_2_ along with TGF-*β*1 and PAF plays an actively suppressing role in the shift from a pro-inflammatory to a more anti-inflammatory phenotype in M*ϕ* that have ingested apoptotic cells.

## 5. Conclusions and Future Perspectives

Compared with pM*ϕ* from uninfected CAPD patients, pM*ϕ* from an infected peritoneal cavity display *ex vivo* an upregulation of production and secretion of pro-inflammatory cytokines and a downregulation of anti-inflammatory mediators. In terms of polarized macrophage activation, these findings show that during infectious peritonitis the pM*ϕ* population is on average shifted to a M*ϕ*1 phenotype. In the above-mentioned studies, the cells were collected when the first signs and symptoms of peritonitis became manifest, that is, before antibiotic treatment was started. Following successful treatment, signs and symptoms improve within a few days. *Ex vivo* studies with effluents could also provide an unique opportunity to follow up human pM*ϕ* and other leukocytes during the resolution phase, set in motion after antibiotics have brought about reduction and elimination of microbes. What changes do pM*ϕ* and other leukocytes undergo in the recovery phase during the shift from M*ϕ*1 to a more typical M*ϕ*2 profile? What is the time course and how long do M*ϕ*1 features persist? Using current techniques including transcriptional profiling, proteomics and flow cytometry, a better understanding of the regulation of infection-induced inflammatory reactions in humans may be achieved. The findings of the comparative studies on cytokine release from pM*ϕ* from an infection-free and infectious environment are in line with the postulate that *in vivo* M*ϕ*1 and M*ϕ*2 are extremes of a wide spectrum of phenotypes. Yet, the fact that M*ϕ*2 may increase by local proliferation rather than by recruitment, as recently found in experimental studies, may have important implications for the way we look at the pathogenesis and therapy of chronic inflammatory disorders, if this interesting discovery also applies in humans [13, 50]. Severe fibrosis and neoangiogenesis of the peritoneum are the histological hallmarks of encapsulating peritoneal sclerosis (EPS), a rare but serious complication of long-term CAPD [51–56]. Etiology and pathogenesis are incompletely understood, but EPS may be conceived as an extreme example of type 2 inflammation. Histological studies and *ex vivo* studies of pM*ϕ* from peritoneal effluents, assuming they are representative of peritoneal tissue M*ϕ*, may help to gain a better understanding of this complication.
